# Contribution of radiation education to anxiety reduction among Fukushima Daiichi Nuclear Power Plant workers: a cross sectional study using a text mining method

**DOI:** 10.1093/jrr/rrab101

**Published:** 2021-11-01

**Authors:** Ryuji Okazaki, Kenichi Satoh, Arifumi Hasegawa, Naoki Matsuda, Takaaki Kato, Reiko Kanda, Yoshiya Shimada, Takuya Hayashi, Masaoki Kohzaki, Kosuke Mafune, Koji Mori

**Keywords:** questionnaire survey, radiation knowledge, radiation anxiety, Fukushima Daiichi Nuclear Power Plant (FDNPP) workers, text mining methods

## Abstract

The purpose of this study is to investigate the frequency of education, knowledge of radiation and workplace anxiety of Fukushima Daiichi Nuclear Power Plant (FDNPP) workers and to analyze what type of words are used for anxiety with a text mining method. An original questionnaire survey was given to FDNPP workers, and a text mining method was used to extract information from free-entry fields. The questionnaires were collected from 1135 workers (response rate: 70.8%). It was found that when workers receive education on radiation, the increased knowledge helps to reduce their anxiety. Among the 1135 workers, 92 of 127 completed the free-entry field with valid entries. Seventy-one words were extracted by the text mining method. The words used differed depending on the degree of anxiety. The text mining method revealed information about the presence or absence of radiation anxiety and the subjects’ working environment and background.

## INTRODUCTION

As a result of the Fukushima Daiichi Nuclear Power Plant (FDNPP) accident in 2011, 174 FDNPP workers were exposed to more than 100 mSv of radiation [1, 2]. It has been reported that most of the exposure was internal [1, 2]. No workers died from the acute effects of radiation. Emergency workers have concerns about the late effects of radiation. According to the annual questionnaire by Tokyo Electric Power Company Holdings, Incorporated (TEPCO), approximately 30% of the workers still have anxiety about radiation [3]. Studies have shown that people who worked during the Chernobyl nuclear accident have long-term mental health problems [4]. Therefore, support to alleviate anxiety about radiation for FDNPP workers is crucial.

According to the Ordinance on the Prevention of Ionizing Radiation Hazards of the Ministry of Health, Labour and Welfare, to work in the FDNPP, new workers must have the new worker education, ‘a’ education (radiation basic knowledge), and ‘b’ education (practical knowledge) and must take a special FDNPP course. The contents of the course include guidelines for safety management measures at TEPCO’s FDNPP (30 min), knowledge about nuclear fuel material or spent fuel and those contaminated by it (30 min), knowledge of working methods in nuclear reactor facilities (1 hour and 30 min), knowledge of the structure and handling of equipment related to nuclear reactor facilities (1 hour and 30 min), related laws and regulations (1 hour) and the biological effects of radiation (30 min). Furthermore, workers must receive special education for work, such as decontamination and special education for work at specific doses (4 hours). Although considerable education about systems and protection is provided, the courses only allocate 30 minutes to cover the biological effects of radiation. According to the Beck Anxiety Inventory score, which focuses primarily on the physical symptoms of anxiety, people who work with radiation are significantly more anxious than non-radiation workers [5]. Operation leaders who are responsible for decontamination workers without radiation knowledge have been found to have increased anxiety and need educational support [6]. To reduce radiation anxiety, workers may need to spend more time on radiation education.

However, there are no reports on the relationship between education and the anxiety of FDNPP workers. This study investigated the frequency of radiation education (previously received by workers, including education on laws on radiation regulations), radiation knowledge and current state of radiation anxiety using a questionnaire for FDNPP workers. Moreover, to investigate whether the anxiety of FDNPP workers is solely due to radiation or other causes (e.g. work adaptation, severe results of radiation anxiety, work motivation), the text in the free text box was analyzed using the text mining method.

## MATERIALS AND METHODS

### Study design and participants

We sent original questionnaires to 1602 FDNPP workers in November 2016. Among the 1602 workers, 104 were from TEPCO and 1498 were from cooperative companies (316 from the primary contractor and 1182 from another contractor). The questionnaire survey was administered anonymously. We considered responses to the survey as signifying the respondents’ consent to participate. The questionnaire survey was approved by TEPCO. The respondents read an explanation of the survey and understood that participation was voluntary.

The study was conducted with approval from the Conflict of Interest Committee (no. 300010) and the Ethics Committee (no. H28–140) of the University of Occupational and Environmental Health, Japan.

### The original questionnaire

We developed and prepared original questionnaires on radiation education, knowledge and anxiety (supplemental data). Each category had four of choices. Radiation education included education that workers had previously received. The frequency of education was identified as: (i) once, (ii) twice, (iii) three times, and (iv) four times or more. The degree of radiation knowledge was identified as: (i) quite high, (ii) a little, (iii) not so much, and (iv) none. The degree of workplace anxiety was identified as: (i) very anxious, (ii) anxious, (iii) slightly anxious, and (iv) not anxious.

In addition, a free-entry field was also provided in the questionnaire. To answer the field, it was described as follows: ‘Finally, we would appreciate it if you could give us your frank opinions for the future. We would appreciate it if you could write them down.’ This time, in order to find out the background of the workplace anxiety, we do not ask directly about anxiety in the free-entry field.

### Data analysis

All statistical analyses were performed using R (The R Foundation for Statistical Computing, Vienna, Austria, version 4.0.3). To perform the correspondence analysis, we used the MASS library. To visualize the results of the correspondence analysis, the text plot function of the word cloud library was used to avoid overlapping text characters.

### Analysis of the frequency of education, workers’ knowledge about radiation and workplace anxiety

A cross-tabulation was performed to test independence (Fisher’s exact test). A p-value of 0.05 or less was used to denote statistical significance. Furthermore, residual analysis was performed to determine the frequency of education, workers’ knowledge about radiation and workers’ workplace anxiety. The z value indicates the discrepancy between the observed frequency and the expected value under independence. For example, z = 1.96 means that the frequency is significantly greater than the expected value, and if it is −1.96, it is significantly smaller with a corresponding p-value of 0.05.

### The text mining methods

The text mining methods using the KH coder, a software developed by Koichi Higuchi at Ritsumeikan University in Japan [7, 8], were described previously [9]. The KH coder uses ‘R’ for statistical analysis. From 92 descriptive answers in the free-entry fields in the original questionnaire, frequent words were extracted using a KH coder based on Japanese morphological analysis. The reason for extracting only nouns is that their meanings are easier to understand than other word classes that are essential for structure, such as verbs and adjectives. To extract approximately 70 to 80 words empirically, we extracted words that were used more than three times [10].

**Fig. 1:**
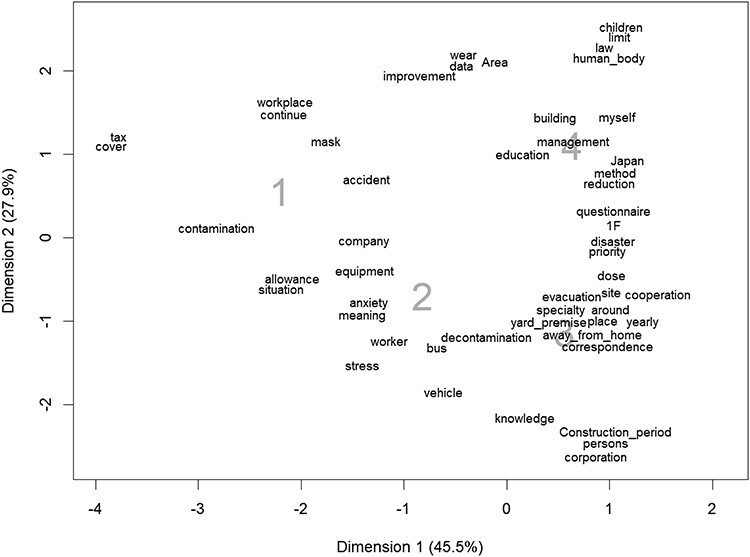
Correspondence analysis between workplace anxiety and words in the free-entry field. The number is the degree of workplace anxiety; 1: very anxious, 2: anxious, 3: slightly anxious and 4: not anxious. The X and Y axis indicates the contribution rate.

Correspondence analysis determines the relative relationship between workplace anxiety and free-text words based on the data given in the contingency table between the level of workplace anxiety and word frequency. Practically, it is preferable to include approximately 70 words. Words close to the number 1 indicate that workers with a level of workplace anxiety of 1 use the word relatively more often than those with other levels. For example, in Fig. 1, the words ‘company’ and ‘equipment’ are close to the number 1, indicating that these words are used relatively more frequently by workers with a workplace anxiety level of 1. However, these words are also found near the number 2. Therefore, they are also used relatively frequently by workers with a level of 2. The words ‘tax’ and ‘cover’ are located behind the number 1, closer to 1 than any other number and farther away from the other numbers. Therefore, these words are characteristic of Level 1. Correspondence analysis is a dimension reduction method similar to principal component analysis, so the amount of information on the two-dimensional plane can be output as the contribution rate. A good analysis result is considered when the total contribution rate of the first axis and the second axis is 70% or more. To decide how many dimensions to retain, rules of thumb similar to those used in PCA and FA are applied. More precisely, commonly used rules recommend that the number of dimensions retained represent 70% or more of the inertia [11].

The text mining method is a powerful tool that can quickly extract important information from a vast amount of biomedical literature [12]. Without specialized knowledge about the text mining method, performing sufficient analysis may not be possible [12]. The text mining method can be sufficiently analyzed if there are approximately 100 descriptions [11]. In the text mining method, word extraction and cluster dendrogram analysis are standard practices. Because this study was conducted to reduce workplace anxiety, we used a more advanced analysis that included a correspondence analysis involving workplace anxiety and the answers extracted from the free-entry fields.

## RESULTS

The questionnaires were collected from 1135 workers (response rate: 70.8%). Among the 1135 workers, 127 filled out the free-entry field and 92 were valid entries. There was no significant difference in the distribution of basic attributes with or without the free-entry field description (supplemental table). The subject who completed the free-entry field can also be interpreted as a group that represents the whole to some extent. In total, 1120 were male and six were female (nine were non-answers). With regard to age, 116 workers were in their twenties, 221 were in their thirties, 352 were in their forties, 293 were in their fifties and 85 were in their sixties and above (68 were non-answers). The mean age was 44.7 ± 10.8 years (range 18–70 years).

Two of each of the three categories (frequency of education, knowledge of radiation and workplace anxiety) were analyzed using Fisher’s exact test and residual analysis (Tables 1–3). No significant relationship was observed between the frequency of education and workplace anxiety (p = 0.152). Significant relationships were found between knowledge about radiation and workplace anxiety (p = 0.003) and between the frequency of education and knowledge about radiation (p = 0.001). Since the results of Fisher’s exact test do not indicate which category has a significant difference in the observed values, a residual analysis was performed to clarify this. Data in columns show the number, percentage and p value of residual analysis. Obvious significance was not recognized between the frequency of education and workplace anxiety. Between the frequency of education and radiation knowledge and between radiation knowledge and workplace anxiety, some columns show significance.

**Table 1:** Relationship between frequency of education and workplace anxiety

		workplace anxiety			
		1: very anxious	2: anxious	3: slightly anxious	4: not anxious
frequency of education	once	3, 1.686, 0.092	2, −0.457, 0.648	5, 0.626, 0.531	2, −1.338, 0.181
	twice	4, 1.483, 0.138	2, −1.439, 0.150	7, 0.140, 0.889	7, 0.140, 0.889
	three times	0, −0.961, 0.336	4, 2.363, 0.018	2, −0.298, 0.765	1, −1.130, 0.258
	four times or more	3, −1.871, 0.061	12, 0.245, 0.807	17, −0.383, 0.702	21, 1.402, 0.161

p value; 0.152

The 4×4 cross-tabulation for both of nominal scales was created to test independence. These data in the columns shows frequency, z value (the adjusted standardized residual), and p value of residual analysis. The p value outside Fisher’s exact test.

**Table 2:** Relationship between frequency of education and knowledge of radiation

radiation knowledge					
		1: quite high	2: a little	3: not so much	4: none
frequency of education	once	1, −1.714, 0.086	5, −1.113, 0.266	5, 3.402, 0.001	1, 1.569, 0.117
	twice	2, −2.148, 0.032	14, 1.374, 0.169	3, 0.474, 0.635	1, 0.980, 0.327
	three times	2, −0.047, 0.963	4, 0.034, 0.972	1, 0.198, 0.843	0, −0.410, 0.682
	four times or more	22, 2.986, 0.003	29, −0.407, 0.684	2, −2.820, 0.005	0, −1.667, 0.096

The 4×4 cross-tabulation for both of nominal scales was created to test independence. These data in the columns shows frequency, z value (the adjusted standardized residual), and p value of residual analysis. The p value outside Fisher’s exact test.

**Table 3:** Relationship between knowledge of radiation and workplace anxiety

		workplace anxiety			
		1: very anxious	2: anxious	3: slightly anxious	4: not anxious
radiation knowledge	1: quite high	2, −0.688, 0.492	6, 0.072, 0.942	4, −2.469, 0.014	15, 2.859, 0.004
	2: a little	4, −1.116, 0.264	14, 1.374, 0.169	19, 0.658, 0.511	15, −1.122, 0.262
	3: not so much	3, 1.863, 0.062	0, −1.863, 0.062	7, 2.239, 0.025	1, −1.840, 0.066
	4: none	1, 1.798, 0.072	0, −0.754, 0.451	1, 0.493, 0.622	0, −1.019, 0.308

p value; 0.003

The 4×4 cross-tabulation for both of nominal scales was created to test independence. These data in the columns shows frequency, z value (the adjusted standardized residual), and p value of residual analysis. The p value outside Fisher’s exact test.

In Table 4, words used three times or more were extracted from the free-entry field of the questionnaires. Seventy-one words were extracted. The words used 10 times or more were ‘radiation’ (37 times), ‘work’ (36 times), ‘Fukushima’ (22 times), ‘exposure’ (19 times), ‘management’ (17 times), ‘1F’ (abbreviation for FDNPP; 13 times), ‘job’ (12 times), ‘dose’ (12 times) and ‘abolition’ (10 times).

Figure 1 shows the correspondence analysis between workplace anxiety and the words in the free-entry field. Respondents with high workplace anxiety used the words ‘contamination,’ ‘masks,’ and ‘accidents,’ among others, relatively frequently, whereas those with low anxiety used the words ‘children,’ ‘education,’ ‘reduction’ and ‘management,’ among others.

## DISCUSSION

For FDNPP workers, there are few reports analyzing the relationship between radiation anxiety and education, and there is no analysis using the text mining method. As expected from the results of this study, the higher the frequency of education, the more knowledge and the less anxiety FDNPP workers have. The text mining method makes it possible to identify opinions related to the power plant premises, radiation control and life as well as whether the workers had radiation anxiety.

Since May 2015, the FDNPP premises have been divided into green, yellow and red zones. A disposable mask is used in the green zone, a half-face mask is used in the yellow zone and a full-face mask is used in the red zone. The red zone has the highest radiation dose. In this questionnaire, we compared the values of the General Health Questionnaire (GHQ)-12 by industry and by zone. Most workers were in the normal range, but in the TEPCO group companies, workers in the red zone had a median score of 5. Even healthy people could score 4–5 when stressed. Even though the anxiety level was high, it was much lower than 4–5 (Supplementary Data 1).

**Table 4:** The words extracted from the free-entry fields of the questionnaire

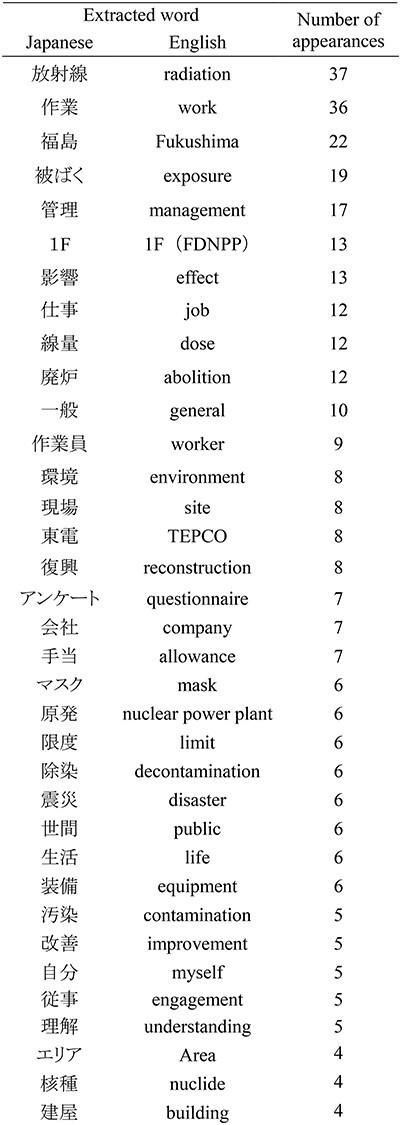
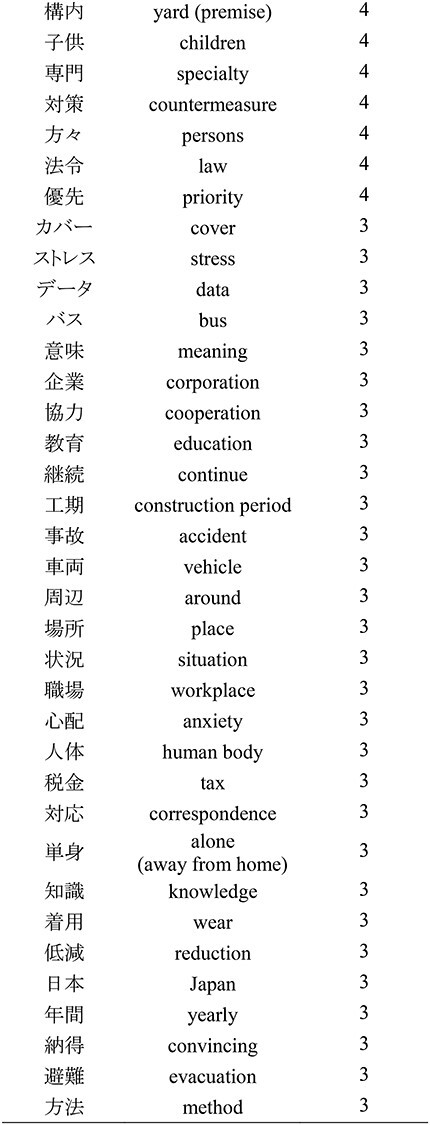

Tables 1–3 shows the statistical relationships between the three categories (frequency of education, radiation knowledge and workplace anxiety). Fisher’s exact test was used to test the independence of the items in the rows and columns of the contingency table. The results showed that the null hypothesis of independence between frequency of education and workplace anxiety was not rejected at the 5% level of significance. On the other hand, the null hypothesis for the table of education and radiation knowledge and for radiation knowledge and workplace anxiety was rejected at the 5% level of significance, indicating that they are not independent. Next, we conducted a residual analysis to determine the specific trends. The adjusted standardized residual (z value) of the cell (once, 4: none) between education and radiation knowledge was 1.569, which is positively large. In other words, when the frequency of education is low, the number of people without radiation knowledge is high. However, the p value is 0.117, which is more than 5% and it is not statistically significant. On the other hand, in the cell (four times or more, 1: quite high), z = 2.986, p = 0.003, indicating that workers tend to have high radiation knowledge if the frequency of education is high. In the same way, the test of independence between radiation knowledge and workplace anxiety was rejected at the 5% level of significance. The residual analysis showed, for example, that z = 2.859, p = 0.004 in the cell (1: quite high, 4: not anxious), indicating that workers with high radiation knowledge do not feel workplace anxiety. Overall, education increases knowledge and reduces anxiety.

Uncertain information, such as information from social networking sites (SNSs), increases anxiety, but information from Nippon Hoso Kyokai (NHK, Japan Broadcasting Corporation) reduces anxiety in Fukushima [13], suggesting the importance of correct information. We conducted a previous evaluation using path analysis to determine what type of knowledge reduced anxiety in four categories (effects on the human body, radiation protection, radiation measurement and evaluation of exposure dose) [14]. Increased knowledge of the effects of radiation on the human body and radiation protection reduced workplace anxiety. However, knowledge on how to measure radiation increased workplace anxiety, possibly because radiation measurements might reveal high radiation doses and the risks.

In statistical text analysis, a maximum of approximately 50 words are empirically used in word visualization [15, 16]. In this study, 71 words were extracted. If two or more words were extracted, there were 130 words, and if four or more words were extracted, 42 words were obtained. The word ‘radiation’ occurred most frequently in the answers extracted from the free-entry fields. In addition, ‘work’ and ‘Fukushima’ were the words that were used often (Table 4).

Correspondence analysis is a tool that can easily show the relationships of various categories on a graphic display [12]. Highly anxious workers (degree of workplace anxiety: 1) used the words ‘mask,’ ‘contamination’, ‘accident,’ ‘workplace,’ ‘continue,’ ‘cover’ and ‘tax’ (Figure 1). The following is a summary of the comments of a worker who is very anxious (workplace anxiety 1). ‘Areas with and without masks are mixed in FDNPP. I’m worried because I feel anxiety because I don’t know where the contamination is.’ The workers described radiation anxiety as well as dissatisfaction with the work environment and anxiety in life. Workers without anxiety (degree of workplace anxiety: 4) mentioned no anxiety about radiation. Some expressed willingness to work and a wealth of knowledge regarding radiation. It is suggested that workers with low radiation anxiety had high radiation knowledge because they used specialized words; however, workers with high radiation anxiety used many words related to real life rather than low knowledge [17].

Thirty years after World War II, radiation classes were excluded from compulsory education. Since 2012, lectures on the biological effects of radiation have begun for junior high school students. Little knowledge is imparted in high school and medical school, and students do not acquire detailed knowledge regarding atomic bomb survivors. Legal education before entering the nuclear power plant focuses on radiation safety, handling and management. Since new workers enter the FDNPP every year, their knowledge of radiation may not always be high [14, 18]. In contrast, potential workers at the Chernobyl Nuclear Power Plant must take a 40-hour course and pass a national examination before working [19], and none of these workers reported radiation anxiety [19]. This may also be because the education involves educational content such as self-management for stress, not only knowledge of radiation. In the event of a disaster, the following five interventions are known principles; (i) a sense of safety, (ii) calming, (iii) a sense of self- and community efficacy, (iv) connectedness, and (v) hope [20]. Education aimed at improving stress coping may also be needed. Alternatively, there may be differences in motivation for jobs compared to Fukushima. Japanese individuals obtain considerable radiation information from SNSs, but that information increases anxiety [13]. Even though it has been several years since the accident, it is difficult to say that knowledge has improved. The importance of radiation education has been described in many papers after the FDNPP accident [21–24]. One of these studies explains the importance of sustainable and continuous radiation education [24]. Moreover, education related to mental health is important for radiation decontamination workers to reduce their anxiety about radiation [17]. Previously, we reported that there was a considerable difference in the influence of anxiety factors on the human body [25]. The text mining method can show not only what was written but also what kind of person wrote it and the relationship of the text to their background, by integrating the results of text mining and other information, such as the degree of anxiety. This study suggests that it is necessary not only to improve knowledge through education and reduce anxiety but also to understand the background of workers’ anxiety, which may be linked to risk communication.

In conclusion, education is important to reduce radiation anxiety and to develop effective education, and knowledge of what kind of anxiety workers have and their background is important. Knowledge about radiation in this study is limited in that it is subjective, and not objective knowledge. Moreover, since this is a cross-sectional study, a causal relationship cannot be estimated. It is necessary to conduct a longitudinal study in the future.

## Supplementary Material

supplementary_rrab101

Supplemental_table_rrab101
