# Supplementary material for: Contribution of radiation education to anxiety reduction among Fukushima Daiichi Nuclear Power Plant workers: a cross sectional study using a text mining method
Source: J Radiat Res. 2021 Nov 1;63(1):44–50. doi: 10.1093/jrr/rrab101 (PMC8776688; doi:10.1093/jrr/rrab101)
Supplement: supplementary_rrab101 [file supplementary_rrab101.pptx]

## Slide 1
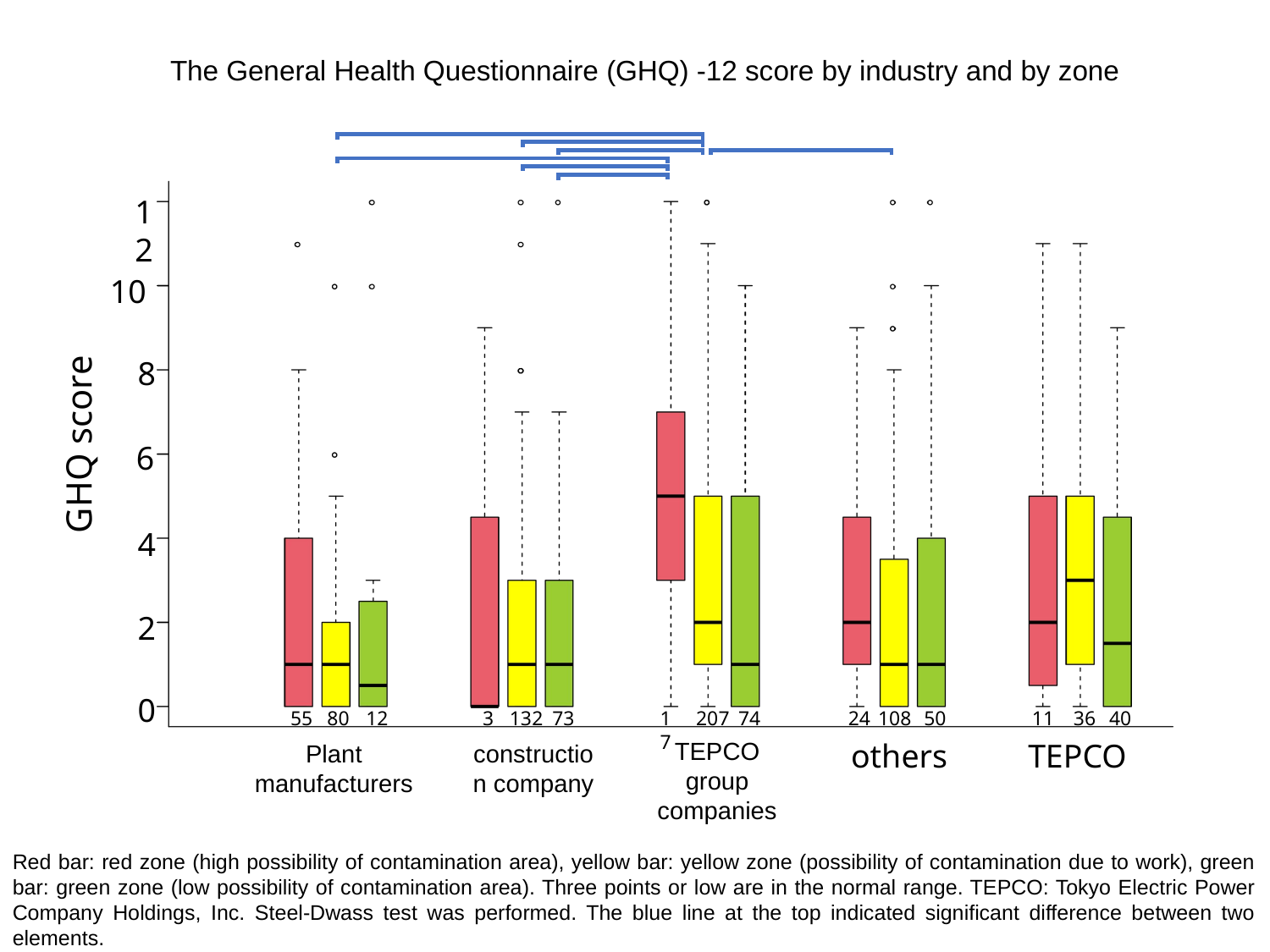

The General Health Questionnaire (GHQ) -12 score by industry and by zone
12
10
8
6
4
2
0
50
55
80
12
3
132
73
17
207
74
24
108
11
36
40
TEPCO
group companies
others
TEPCO
Plant manufacturers
construction company
GHQ score
Red bar: red zone (high possibility of contamination area), yellow bar: yellow zone (possibility of contamination due to work), green bar: green zone (low possibility of contamination area). Three points or low are in the normal range. TEPCO: Tokyo Electric Power Company Holdings, Inc. Steel-Dwass test was performed. The blue line at the top indicated significant difference between two elements.
