# Supplementary material for: Contribution of radiation education to anxiety reduction among Fukushima Daiichi Nuclear Power Plant workers: a cross sectional study using a text mining method
Source: J Radiat Res. 2021 Nov 1;63(1):44–50. doi: 10.1093/jrr/rrab101 (PMC8776688; doi:10.1093/jrr/rrab101)
Supplement: Supplemental_table_rrab101 [file supplemental_table_rrab101.docx]

Supplemental table: Attribute evaluation with or without free-entry field description

|  | with the free-entry field description | | | |  | without the free-entry field description | | | |  | p | |  |  |
| --- | --- | --- | --- | --- | --- | --- | --- | --- | --- | --- | --- | --- | --- | --- |
|  | N | (%) | M | (SD) |  | N | (%) | M | (SD) |  |  | |  |  |
| man | 125 | (11.2) |  |  |  | 995 | (88.8) |  |  |  | .670 | | # |  |
| woman | 1 | (16.7) |  |  |  | 5 | (83.3) |  |  |  |  | |  |  |
|  |  |  |  |  |  |  |  |  |  |  |  | |  |  |
| age |  |  | 46.1 | (10.3) |  |  |  | 44.5 | (10.9) |  | .131 | | $ |  |
| 10- | 0 | (0.0) |  |  |  | 2 | (100.0) |  |  |  | .129 | | # |  |
| 20- | 9 | (7.8) |  |  |  | 106 | (92.2) |  |  |  |  | |  |  |
| 30- | 19 | (8.6) |  |  |  | 202 | (91.4) |  |  |  |  | |  |  |
| 40- | 50 | (14.2) |  |  |  | 302 | (85.8) |  |  |  |  | |  |  |
| 50- | 28 | (9.6) |  |  |  | 265 | (90.4) |  |  |  |  | |  |  |
| 60- | 13 | (15.3) |  |  |  | 72 | (84.7) |  |  |  |  | |  |  |
|  |  |  |  |  |  |  |  |  |  |  |  | |  |  |
| married | 80 | (11.4) |  |  |  | 620 | (88.6) |  |  |  | | .454 | # | |
| single | 40 | (10.3) |  |  |  | 347 | (89.7) |  |  |  | |  |  | |
| other | 7 | (16.7) |  |  |  | 35 | (83.3) |  |  |  | |  |  | |
| live |  |  |  |  |  |  |  |  |  |  | |  |  | |
| out of Fukushima | 61 | (13.5) |  |  |  | 391 | (86.5) |  |  |  | | .050 | # | |
| in Fukushima | 65 | (9.7) |  |  |  | 603 | (90.3) |  |  |  | |  |  | |
| Industry |  |  |  |  |  |  |  |  |  |  | |  |  | |
| Plant manufacturers | 24 | (15.1) |  |  |  | 135 | (84.9) |  |  |  | | .316 | # | |
| construction company | 29 | (12.2) |  |  |  | 208 | (87.8) |  |  |  | |  |  | |
| TEPCO group companies | 37 | (10.2) |  |  |  | 325 | (89.8) |  |  |  | |  |  | |
| others | 27 | (10.9) |  |  |  | 220 | (89.1) |  |  |  | |  |  | |
| TEPCO | 7 | (7.1) |  |  |  | 92 | (92.9) |  |  |  | |  |  | |
| Years of work experience | | |  |  |  |  |  |  |  |  | |  |  | |
| <1 year | 15 | (12.5) |  |  |  | 105 | (87.5) |  |  |  | | .821 | # | |
| 1 ～ 5 years | 40 | (10.1) |  |  |  | 357 | (89.9) |  |  |  | |  |  | |
| 5 ～ 10 years | 17 | (10.7) |  |  |  | 142 | (89.3) |  |  |  | |  |  | |
| >10 years | 52 | (11.8) |  |  |  | 388 | (88.2) |  |  |  | |  |  | |
| Years of work in FDNPP | | |  |  |  |  |  |  |  |  | |  |  | |
| none | 3 | (15.0) |  |  |  | 17 | (85.0) |  |  |  | | .119 | # | |
| <1 year | 22 | (15.5) |  |  |  | 120 | (84.5) |  |  |  | |  |  | |
| 1 ～ 3 years | 49 | (12.4) |  |  |  | 345 | (87.6) |  |  |  | |  |  | |
| 3 ～ 5 years | 15 | (7.2) |  |  |  | 193 | (92.8) |  |  |  | |  |  | |
| >5 years | 37 | (10.1) |  |  |  | 330 | (89.9) |  |  |  | |  |  | |
| Exposed dose |  |  |  |  |  |  |  |  |  |  | |  |  | |
| Not know | 16 | (17.4) |  |  |  | 76 | (82.6) |  |  |  | | .459 | # | |
| <1mSv | 18 | (8.3) |  |  |  | 200 | (91.7) |  |  |  | |  |  | |
| 1～5mSv | 18 | (13.3) |  |  |  | 117 | (86.7) |  |  |  | |  |  | |
| 5～10mSv | 14 | (15.1) |  |  |  | 79 | (84.9) |  |  |  | |  |  | |
| 10～20mSv | 14 | (12.2) |  |  |  | 101 | (87.8) |  |  |  | |  |  | |
| 20～50mSv | 22 | (10.0) |  |  |  | 198 | (90.0) |  |  |  | |  |  | |
| 50～75mSv | 15 | (11.6) |  |  |  | 114 | (88.4) |  |  |  | |  |  | |
| 75～100mSv | 5 | (6.8) |  |  |  | 69 | (93.2) |  |  |  | |  |  | |
| 100～150mSv | 2 | (11.1) |  |  |  | 16 | (88.9) |  |  |  | |  |  | |
| 150～200mSv | 0 | (0.0) |  |  |  | 2 | (100.0) |  |  |  | |  |  | |
| >200mSv | 0 | (0.0) |  |  |  | 2 | (100.0) |  |  |  | |  |  | |
|  |  |  |  |  |  |  |  |  |  |  | |  |  | |
| Workplace anxiety | |  | 2.9 | (0.9) |  |  |  | 2.8 | (0.9) |  | | .536 | $ | |
| very anxious | 11 | (16.4) |  |  |  | 56 | (83.6) |  |  |  | | .223 | # | |
| anxious | 30 | (9.1) |  |  |  | 298 | (90.9) |  |  |  | |  |  | |
| slightly anxious | 47 | (10.9) |  |  |  | 385 | (89.1) |  |  |  | |  |  | |
| not anxious | 39 | (13.2) |  |  |  | 257 | (86.8) |  |  |  | |  |  | |
| Working zone |  |  |  |  |  |  |  |  |  |  | |  |  | |
| red | 18 | (13.8) |  |  |  | 112 | (86.2) |  |  |  | | .307 | # | |
| yellow | 88 | (11.8) |  |  |  | 659 | (88.2) |  |  |  | | .381 | # | |
| green | 52 | (10.2) |  |  |  | 458 | (89.8) |  |  |  | | .338 | # | |
|  |  |  |  |  |  |  |  |  |  |  | |  |  | |
| # P value for chi square test | | | | $ p value for t-test | | | |  |  |  | |  |  | |
